# Evaluation of Migration, Bioaccessibility, and Dietary Risk of Organophosphate Flame Retardants in Polypropylene Packaging Using a Packaging–Food–Digestion Simulation System

**DOI:** 10.3390/foods15040780

**Published:** 2026-02-21

**Authors:** Fan Shen, Weili Li, Junjian Miao, Shu Liu, Keqiang Lai

**Affiliations:** 1 College of Food Science and Technology, Shanghai Ocean University, No. 999 Hucheng Huan Road, LinGang New City, Shanghai 201306, China; 2 Engineering Research Center of Food Thermal-Processing Technology, Shanghai 201306, China; 3 Technical Center for Industrial Product and Raw Material Inspection and Testing of Shanghai Customs District, Shanghai 200135, China

**Keywords:** OPFRs, migration experiment, in vitro digestion experiment, bioaccessibility, risk assessment

## Abstract

This study systematically investigated the migration behavior, bioaccessibility, and dietary risk of five typical organophosphate flame retardants (OPFRs) in polypropylene (PP) packaging using migration experiments and in vitro simulated digestion. Migration was primarily influenced by molecular structural features, including polarity, volume, and flexibility, and was further modulated by the food matrix composition. Diffusion and partition coefficients effectively characterized the migration patterns of OPFRs in different foods. In vitro digestion results indicated that molecular polarity was the main structural factor affecting bioaccessibility, while food matrix composition significantly influenced the bioaccessibility of all compounds except TnBP. Dietary risk assessment, incorporating bioaccessibility, improved the accuracy of exposure estimation. At a PP incorporation level of 0.1 g/kg, all five OPFRs showed hazard quotient (HQ) values below 1 across all dietary scenarios, indicating acceptable risk. TBOEP and TPPO exhibited relatively higher HQ values, warranting closer attention. The “packaging–food–digestion simulation” system established in this study integrated migration data and bioaccessibility results to represent the exposure process of OPFRs from packaging through food to human digestion and provided a practical basis for risk assessment of packaging additives.

## 1. Introduction

Polypropylene (PP) is commonly used in food packaging because of its good physicochemical properties and low price [1]. However, a single polymer material cannot satisfy different performance requirements such as flame retardancy, heat resistance, and oxidation resistance at the same time. Therefore, different kinds of functional additives are usually added in the production process to improve these properties. With the worldwide ban and prohibition of brominated flame retardants [2], organophosphate flame retardants (OPFRs) are widely used in PP packaging to improve flame retardancy because of their low smoke emission and relatively low toxicity [3,4].

Previous studies have reported that the dietary intake is the main way OPFRs enter the human body [5,6]. Owing to the existence of phosphate ester groups and aromatic rings in the molecular structure of OPFRs, these compounds generally show high chemical stability and lipophilicity and can accumulate in the body. Long-term exposure to OPFRs may cause neurotoxicity, endocrine disruption, and reproductive adverse effects [7,8,9,10]. Recent risk-oriented reviews have further consolidated current evidence on OPFR exposure pathways and associated health effects, highlighting their widespread occurrence and multiple human exposure routes [11]. In recent years, the migration of harmful substances from packaging materials into food has received increasing attention. At present, a large number of substances have been widely studied, such as heavy metals, mineral oils [12], bisphenol compounds [13], chlorinated paraffins [14,15], phthalates [16], and perfluorinated compounds [17]. Compared with the above well-known pollutants, knowledge of the migration behavior of OPFRs in different food systems remains limited, and regulatory provisions governing their migration from food contact materials (FCMs) are still scarce.

In recent years, bioaccessibility (BA) was gradually incorporated into dietary exposure and risk assessment frameworks and has been widely applied in studies of heavy metals and pesticide residues [18,19,20,21]. BA is typically determined using in vitro digestion simulation systems. Compared with traditional dietary exposure that assumes complete absorption of contaminants by the human body, incorporating BA provides a more realistic estimate of the fraction released from the food matrix and available for intestinal absorption, thereby improving the scientific validity of dietary exposure. However, in studies of food contact materials, particularly those focusing on additives, BA was rarely considered.

Based on Wang et al.’s study on the migration of OPFRs in Chinese tea packaging materials [22], this research selected the five most common OPFRs in PP packaging materials as subjects: triphenyl phosphate (TPhP), 2-ethylhexyl diphenyl phosphate (EHDPP), tris(2-butoxyethyl) phosphate (TBOEP), tri-n-butyl phosphate (TnBP), and triphenylphosphine oxide (TPPO). Although TPPO is structurally a phosphine oxide rather than a phosphate ester, it has been increasingly included together with conventional OPFRs as an OPFR-related contaminant in recent high-quality environmental and food-contact related studies and has been reported to occur in food and food-contact materials with measurable human exposure relevance. Therefore, TPPO was included in this study following recent OPFR research practice [22,23,24].

Unlike most previous studies focused mainly on migration using food simulants or on either migration or digestion separately. This study constructed a “packaging–food–digestion simulation” research system to: (1) systematically evaluate the migration characteristics of five typical OPFRs in different foods from the perspectives of molecular structure and food matrix composition; (2) study the differences in bioaccessibility of OPFRs in different foods through a standardized in vitro digestion model; (3) develop a dietary risk assessment framework that integrates migration ratio (MR) and bioaccessibility results to improve the accuracy of OPFR exposure assessment. This research system was designed within the FCM testing and food classification framework, aiming to provide reference information for the development of future regulatory systems related to OPFRs.

## 2. Materials and Methods

### 2.1. Reagents and Materials

Triphenyl phosphate (TPhP, 98.0%), 2-ethylhexyl diphenyl phosphate (EHDPP, 97.0%), tri(2-butoxyethyl) phosphate (TBOEP, 95.0%), tri-n-butyl phosphate (TnBP, 99.0%), and triphenyl phosphine oxide (TPPO, 98.0%) were purchased from Aladdin Reagent Co., Ltd. (Shanghai, China). Isotopically labeled internal standards Triphenyl phosphate-d15 (TPhP-d15, 98.0%) and tri-n-butyl phosphate-d27 (TnBP-d27, 97.3%) were purchased from Shanghai Anpu Experimental Technology Co., Ltd. (Shanghai, China). Salivary amylase solution (10,000 U/g) was purchased from Feijing Biotechnology Co., Ltd. (Beijing, China); pepsin (derived from porcine gastric mucosa, >3000 U/mg) and lipase (20,000 U/g) were purchased from McLean Biochemical Technology Co., Ltd. (Shanghai, China); Trypsin (derived from porcine pancreas, 1:250 dilution), pancreatic lipase (derived from porcine pancreas, 15–35 U/mg), and porcine bile salts (cholic acid content > 60%) were all purchased from Yuanye Biotechnology Co., Ltd. (Shanghai, China). Sodium chloride (NaCl), potassium dihydrogen phosphate (KH_2_PO_4_), sodium bicarbonate (NaHCO_3_), magnesium chloride hexahydrate (MgCl_2_·6H_2_O), ammonium carbonate ((NH_4_)_2_CO_3_), hydrochloric acid (HCl), and calcium chloride dihydrate (CaCl_2_·2H_2_O) were purchased from Sinopharm Chemical Reagent Co., Ltd. (Shanghai, China), all with analytical grade purity or higher. HPLC-grade acetonitrile (99.99%) and formic acid (99.0%) were purchased from J&K Scientific Technology Co., Ltd. (Beijing, China). Polypropylene (PP) was supplied as granular pellets (Model: BC03C, density 0.90 g/cm^3^) from Japan Corporation. Ultrapure water was provided by a Milli-Q ultrapure water system (Millipore, MA, USA) with a resistivity of 18.3 (MΩ/cm).

### 2.2. Preparation of Positive Samples

Polypropylene (PP) films containing target substances were prepared via melt blending and extrusion-calendering processes, with the following specific steps: First, the target substances were added to the PP matrix at an initial concentration of 0.1 g/kg; subsequently, uniform mixing and extrusion were performed using a twin-screw extruder (Hartek Technologies Ltd., Guangzhou, China); Finally, the mixture was calendered into an 80 μm thick film using a three-roll calender. The speed of the extrusion screw was 80 rpm and the sequentially set temperatures of the barrel were as follows: 165 °C (Zone 1), 170 °C (Zone 2), 175 °C (Zone 3), 180 °C (Zones 4–5), 185 °C (Zone 6), 180 °C (Zone 7), 175 °C (Zone 8), 170 °C (Screen Zone), and 170 °C (Die Zones 1–3). The speed of the calendering line was 3.3 m/min; the front, middle, and rear roll speeds were all 2.7 m/min. The resulting film was stored under dry conditions for subsequent migration testing.

To evaluate the initial homogeneity of the target compounds in the prepared PP films, nine positions were randomly selected from the films and analyzed after solvent extraction. Because small variations in the mass of the film pieces are unavoidable, the homogeneity assessment was based on mass-normalized values. Specifically, the measured amounts were normalized to the mass of each film piece, and only minor variations were observed among different sampling positions, indicating a homogeneous initial distribution of the target compounds. During UHPLC–MS analysis of the film extracts, no additional chromatographic peaks corresponding to degradation products were observed, suggesting that no significant thermal degradation occurred under the applied processing conditions.

Food samples with target compounds were prepared by the spiking method with standard solutions. The procedure was as follows: A mixed standard solution (1 μg/mL) containing five OPFRs was added to the blank food sample. The final concentration of the five OPFRs was 100 ng/mL in the food sample. The sample was mixed well and used for in vitro simulated digestion experiments.

### 2.3. Migration Experiment

The selection of food samples was designed according to the classification principle in GB 31604.1-2023 [25] and finally determined as follows. The real foods selected in this study were classified to match the food categories defined for food contact material testing in GB 31604.1-2023 [25]: aqueous food (Eastern Leaf, Jasmine Tea, Soda Water), acidic food (Vinegar, Beer, Cooking Wine), and dairy product/moderately alcoholic food (Soy Milk, Baijiu, Coconut Milk). Baijiu was included to represent a conservative (worst-case) exposure scenario. All food items were purchased from local Chinese supermarkets and used within their expiration dates.

Migration tests were performed at 40 °C for contact times ranging from 0.5 to 72 h. The ratio of the surface area of food contact material and food was controlled at 6 dm^2^/L. In accordance with GB 5009.156-2016 [26], for fully immersed tests involving films thinner than 0.5 mm, only the single-sided area was calculated.

The Prepared PP films (film thickness approximately 0.08 mm) were cut into 3 cm × 3 cm squares and placed in glass containers. Each container was filled with 15 mL of the corresponding food sample and sealed to ensure complete contact during the migration experiments.

After the migration test, the food sample was filtered to remove the PP film and other impurities. Any volume lost due to evaporation during the test was compensated by adding the same food to bring the sample to 15 mL. Subsequently, 2 mL of the sample was taken, an internal standard was added, the sample was treated with 2 mL of acetonitrile, centrifuge to collect the supernatant, then dilute with water to a final volume of 15 mL. Purified using a C18 solid-phase extraction column (300 mg/3 mL, CNW, Shanghai, China). Finally, the solution was filtered through a 0.22 μm PTFE membrane for UHPLC–MS analysis [27,28,29]. All migration experiments were conducted in triplicate (n = 3), and the results were presented as mean ± standard deviation.

The results obtained can be used to calculate the MR. The formula is as follows:

(1) $$\mathrm{MR}(\%)\;=\;\frac{m_{m}}{m_{i}}\;\times \;100$$

where *m_m_* (ng) represents the mass of the target substance migrating into the food, and *m_i_* (ng) is calculated as the product of the weighed mass of each PP film specimen used in the migration test and the controlled loading level applied during film preparation (0.1 g/kg).

### 2.4. Migration Diffusion Model

The migration process of target substances from polymers into food can generally be divided into three stages: diffusion within the polymer matrix, dissolution/desorption at the polymer-food interface, and diffusion into the food matrix. Furthermore, the migration of target substances from polymers is primarily controlled by diffusion and typically follows Fick’s second law of diffusion (Equation (2)) [30].

(2) $$\frac{\partial C}{\partial t}=D\frac{\partial^{2}C}{\partial x^{2}}$$

Here, *C* represents the concentration of the diffusing substance, *t* denotes the diffusion time, and *x* is the space coordinate measured normal to the section, *D* signifies the diffusion coefficient.

If the migration behavior satisfies the following conditions: (1) the target substance is uniformly distributed within the food contact material; (2) migration occurs only between the food contact material and the food, with no diffusion into air or other media; (3) the food contact material does not undergo swelling. Equation (2) can be solved, yielding the result shown in Equation (3):

(3) $$\frac{M_{F,t}}{M_{F,\infty }}\;=\;1\;-\sum_{n=1}^{\infty }\frac{2\alpha (1\;+\;\alpha )}{1\;+\;\alpha \;+\;\alpha^{2}q_{n}^{2}}exp(-\mathrm{Dt}\frac{q_{n}^{2}}{L^{2}})$$

The symbols *M_F,t_* and *M_F,∞_* represent the migrated amounts of the compound in the food phase at time *t* and at equilibrium, respectively. *L* refers to the film thickness, *α* is a dimensionless parameter related to the distribution of the compound between the polymer and food at equilibrium. The value of *α* is related to the partition coefficient (*K_P/F_*), and the relationship between *α* and *K_P/F_* is shown in Equation (5), where *q_n_* is the n-th nonzero positive root of the equation $\tan q_{n}=\;-\alpha \cdot q_{n}$.

(4) $$K_{P/F}=\frac{C_{P,\infty }}{C_{F,\infty }}$$

(5) $$\alpha =\frac{V^{F}\cdot K_{P/F}}{V^{P}}$$

Here, *V^F^* denotes the volume of the food; *V^P^* denotes the volume of the food contact material, *C_F,∞_* denotes the concentration of the target substance in the food phase when migration reaches equilibrium, *C_P,∞_* denotes the concentration of the target substance in the food contact material phase when migration reaches equilibrium.

### 2.5. In Vitro Simulated Digestion Experiment

Because the migrated concentrations of some compounds (e.g., EHDPP and TnBP) in real food matrices were low (<10 ng/mL), direct in vitro simulated digestion experiments of the migrated food samples would have resulted in target compound levels below the instrumental detection limits. Therefore, in vitro simulated digestion experiments were conducted using spiked food matrices to ensure reliable quantification.

To date, several in vitro digestion simulation models were developed internationally [31,32,33]. The INFOGEST model is currently the most widely used internationally standardized model. It simulates the real digestive process in the oral cavity, stomach and small intestine very well and has good reproducibility and comparability [34]. Therefore, this study used the INFOGEST 2.0 standardized in vitro digestion model to investigate the BA of OPFRs in nine foods during digestion after human ingestion. The activities of the digestive enzyme preparations were verified prior to digestion using a fluorescence-based assay. The measured specific activities of salivary amylase, pepsin, gastric lipase, trypsin and pancreatic lipase were approximately 10, 3000, 20, 250 and 30 U/mg, respectively, which were consistent with the activities specified by the manufacturer. The in vitro digestion procedure followed the standardized INFOGEST 2.0 protocol [34]. The digestive fluids were prepared according to the INFOGEST 2.0 standard model, i.e., simulated salivary fluid (SSF), simulated gastric fluid (SGF), and simulated intestinal fluid (SIF).

The in vitro digestion was performed following the standardized INFOGEST 2.0 static protocol under fed-state conditions. The final activities of digestive enzymes in the digestion mixtures were adjusted to 75 U/mL for salivary α-amylase in the oral phase, 2000 U/mL for pepsin and 60 U/mL for gastric lipase in the gastric phase, and 2000 U/mL for pancreatic lipase in the intestinal phase. Bile salts were added in the intestinal phase to reach a final concentration of 10 mM. The electrolyte compositions of SSF, SGF and SIF, as well as the associated ionic strengths, followed the standardized formulations defined in the INFOGEST 2.0 protocol [34].

After the simulated digestion experiment, the digestive products from the small intestine stage were centrifuged, and the supernatant was collected. This supernatant was purified via C18 solid-phase extraction and filtered through a 0.22 μm PTFE membrane before undergoing quantitative analysis by UHPLC–MS. All in vitro simulated digestion experiments were conducted in triplicate (n = 3), and the results were presented as mean ± standard deviation. The result of this experiment could be used to derive the BA. The equation for calculation was shown below.

(6) $$\mathrm{BA}(\%)=\frac{C_{d}\times \;V_{d}}{C_{s}\;\times \;V_{s}}\;\times \;100$$

where *C_d_* represents the target substance concentration in the digestion solution (ng/mL), *V_d_* denotes the volume of the digestion solution (mL), *C_s_* indicates the spiked concentration in the food sample (ng/mL), and *V_s_* signifies the volume of the food sample (mL).

### 2.6. Mass Spectrometry Analysis

Target compounds were quantified on a PerkinElmer Altus A-30 UHPLC coupled to an AB Sciex 4500 triple-quadrupole mass spectrometer. Separation was achieved on a Waters BEH C18 column (50 × 2.1 mm, 1.7 µm) held at 40 °C. The mobile phases were (A) water with 0.1% formic acid and (B) acetonitrile. Injections were 5 µL and the flow rate was 0.30 mL/min. The gradient was: 0–1 min, 20% B; 1–6 min, 20→40% B; 6–10 min, 40→60% B; 10–13 min, 60→95% B; 13–16 min, held at 95% B; 16.0–16.1 min, 95→20% B; 16.1–20 min, re-equilibrated at 20% B.

Mass spectrometry used ESI in positive mode with the following settings: collision gas pressure of 9 psi, curtain gas set to “medium,” ion-spray voltage 5500 V, source temperature 550 °C, and source gases at 40 psi (GS1) and 50 psi (GS2) [22].

### 2.7. Quality Assurance and Quality Control

To reduce possible background interference, all glassware was sequentially washed with acetonitrile and ultrapure water and thoroughly dried to obtain blank signals below the limit of quantification. The cut packaging films were wrapped in aluminum foil and kept in a low-temperature and dry conditions to avoid exogenous contamination. The samples obtained after migration or in vitro digestion were immediately sealed and stored at −20 °C to prevent sample degradation and loss of target compounds.

Quantification of target compounds was achieved with an internal and external standard method. Standard solutions were prepared and diluted with acetonitrile to obtain standard solutions with concentrations of 1–100 ng/mL. For each calibration curve, the vertical axis was set as the peak area ratio of the target compound to the internal standard, and the horizontal axis was set as concentration. All R^2^ of OPFR calibration curves were above 0.99, and the target compounds were linearly related to the peak area ratio over internal standard. If any sample concentration was higher than the calibration range, the sample solution was diluted before quantification. Three procedural blanks (without addition of target compounds and processed in parallel using the same sample preparation and sealing procedures as the samples) were included in each analytical batch to evaluate background contamination and to correct the measured concentrations of the target compounds. TPhP, TBOEP and TnBP were observed in blank controls, with concentrations of 1.13–1.47 ng/mL, 0.94–1.31 ng/mL and 1.07–1.44 ng/mL, respectively. Any target concentration was corrected by the subtracting appropriate blank value.

During the analytical process, one blank sample was inserted after every nine samples to check the sensitivity of the instrument. Method detection limit (MDL, 0.18–0.62 ng/g) and method quantification limit (MQL, 0.60–2.07 ng/g) were calculated using the signal-to-noise ratio (S/N) method, which represents the concentration levels at which S/N ratios are 3 and 10, respectively.

### 2.8. Risk Assessment

To evaluate the dietary exposure risks of OPFRs through dietary intake, we used the risk assessment framework recommended by the U.S. FDA guidelines [35]. In the traditional approach, the equation for calculating daily intake (EDI, ng/kg bw/day) is shown below [36].

(7) $$EDI=\frac{VC}{BW}$$

where *V* represents the daily intake of the food (kg/day), determined according to the Chinese Dietary Guidelines [37]. This study calculated the Estimated Daily Intake (EDI) using both the average population intake and the 95th percentile intake to characterize typical and high exposure scenarios, respectively. *C* denotes the concentration of the compound in the food (ng/kg), *BW* represents the average body weight (kg) of the target population. Following China’s commonly used exposure assessment reference values, the average adult weight was set at 60 kg.

In this study, the concentration of compounds in food was determined by the final MR. Therefore, the EDI formula can be further rewritten as shown in Equation (8) to enable a sequential analysis of MR, BA, and dietary exposure:

(8) $$EDI\;=\;\frac{V\;\times \;C_{p}{\;\times \;MR}_{f}\;\times \;m_{p}}{\mathrm{BW}\;\times \;100}\times \frac{BA}{100}$$

In this equation, *MR_f_* is the final MR, *C_p_* is the initial content of OPFRs in the PP packaging (µg/g), and *m_p_* is the mass of PP packaging material in contact with 1 kg of food (g/kg). *C_p_* was set at 100 µg/g. Based on S/V = 6 dm^2^/L as specified in GB 5009.156-2016 [26] and considering a film thickness of 80 µm and density of 0.9 g/cm^3^, *m_p_* was calculated to be 4 g/kg.

To further assess potential health risks, this study also calculated the hazard quotient (HQ) using the following equation [38]:

(9) $$HQ\;=\;\frac{EDI}{RfD}$$

The Reference Dose (RfD, ng/kg bw/day) values used for HQ calculation are summarized in Table 1 [39]. When the HQ is less than 1, dietary exposure risk is considered acceptable; when the HQ is greater than or equal to 1, potential health risks may exist.

### 2.9. Statistical Analysis

Differences in mean values were examined using one-way analysis of variance (one-way ANOVA) in SPSS Statistics 27 (IBM Corp., Armonk, NY, USA). The Waller–Duncan multiple-range test was used for post hoc comparisons, and statistical significance was set at *p* < 0.05. All data were checked for normality prior to statistical analysis.

## 3. Results and Discussion

### 3.1. Analysis of Migration Behavior of OPFRs in Different Foods

**Figure 1:**
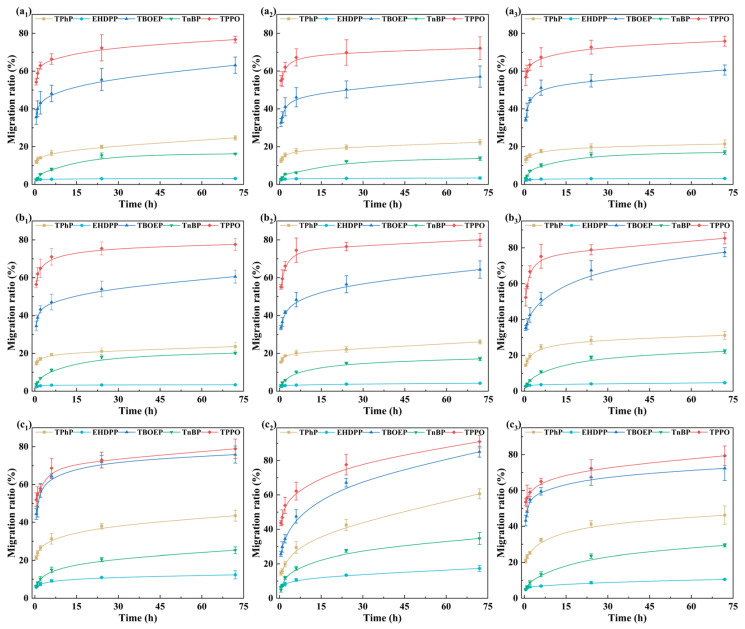
Migration curves of five OPFRs (TPhP, EHDPP, TBOEP, TnBP, and TPPO) from polypropylene films into nine different food matrices: (**a_1_**) Eastern Leaf, (**a_2_**) Jasmine tea, (**a_3_**) Soda water, (**b_1_**) Vinegar, (**b_2_**) Beer, (**b_3_**) Cooking wine, (**c_1_**) Soy milk (**c_2_**) Baijiu, (**c_3_**) Coconut milk. Migration ratios are expressed as a function of time (0–72 h), and the curves were fitted using B-spline functions for visual presentation only. Data is presented as mean ± standard deviation (n = 3).

As shown in Figure 1, the migration behavior of OPFRs in all foods exhibited two different phases, characterized by a rapid increase in the early stage followed by a gradual increase in the later stage. The migration behavior of OPFRs was consistent with the results of Xing et al. for TPhP migration from PP packaging into oily food simulants [40]. Meanwhile, the relative migration ratios of the five OPFRs in different foods consistently followed the order: TPPO > TBOEP > TPhP > TnBP > EHDPP.

These observations suggested that the relative migration performance of OPFRs in foods was primarily governed by their structural characteristics, particularly polarity, molecular volume, and flexibility. In general, smaller molecular volume and higher flexibility facilitate diffusion within the PP matrix, whereas polarity influences both interactions with the polymer and partitioning toward the food phase. For example, TPPO, which exhibited the highest polarity and the smallest molecular volume, showed the strongest migration tendency. TBOEP had slightly lower polarity than TPPO; although it possessed the highest flexibility, its long side chain resulted in a relatively large molecular volume, which could hinder diffusion and thus slightly reduced its migration capacity compared with TPPO. TPhP and TnBP had intermediate values for these structural parameters and therefore showed no pronounced advantages or constraints in migration. In contrast, EHDPP exhibited the lowest polarity, lower flexibility, and larger molecular volume, which markedly restricted its diffusion and resulted in the lowest migration level.

Then, the migration behaviors of three types of food were analyzed separately to explore how food matrices affected the migration behavior.

#### 3.1.1. Analysis of Migration Behavior of OPFRs in Aqueous Foods

**Figure 2:**
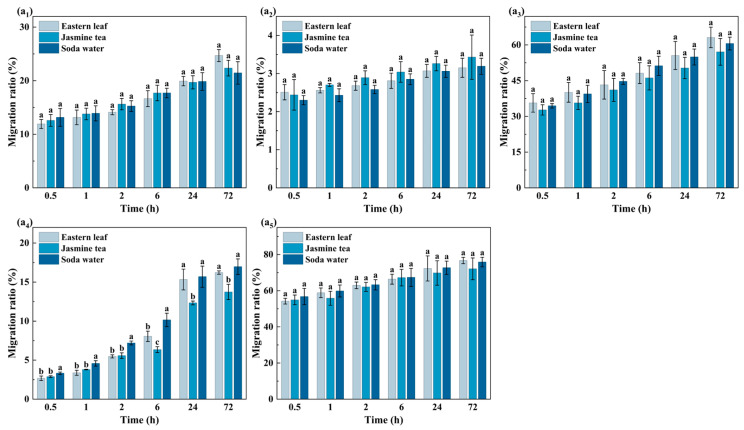
Migration ratios of five OPFRs from PP films into three aqueous food systems (Eastern leaf, Jasmine tea, and Soda water) at different contact times: (**a_1_**) TPhP, (**a_2_**) EHDPP, (**a_3_**) TBOEP, (**a_4_**) TnBP, and (**a_5_**) TPPO. Data is presented as mean ± standard deviation (n = 3). Different lowercase letters above the bars indicate statistically significant differences among different foods at the same contact time for the same compound (*p* < 0.05, Waller–Duncan test).

In order to evaluate the impact of food matrices on migration behavior, aqueous foods with simple matrices and minimal interference were initially selected for analysis. As shown in Figure 2, among the three aqueous foods, the MR of TPPO, TBOEP, TPhP, and EHDPP exhibited minimal variation, indicating that differences among food matrices exerted a relatively small influence on their migration behavior. In contrast, TnBP exhibited significant migration differences across the aqueous food matrices. Specifically, its MR in jasmine tea was markedly lower than in Eastern leaf and soda water. This was because the soluble carbohydrates in Jasmine tea increased the system polarity and further form a hydrogen-bond network, which restrained diffusion behavior. Meanwhile, TnBP molecules had longer alkyl side chains and lower flexibility, and their migration behavior was more sensitive to steric hindrance. Therefore, food matrices enhanced the inhibitory effect on the migration of TnBP.

Generally, the composition of aqueous foods was relatively simple, which had little impact on migration behavior. However, compared with aqueous foods, in the acidic foods containing organic acids or ethanol, the system polarity and matrix environment changed, and the impact on migration behavior was different from that of aqueous foods.

#### 3.1.2. Analysis of Migration Behavior of OPFRs in Acidic Foods

**Figure 3:**
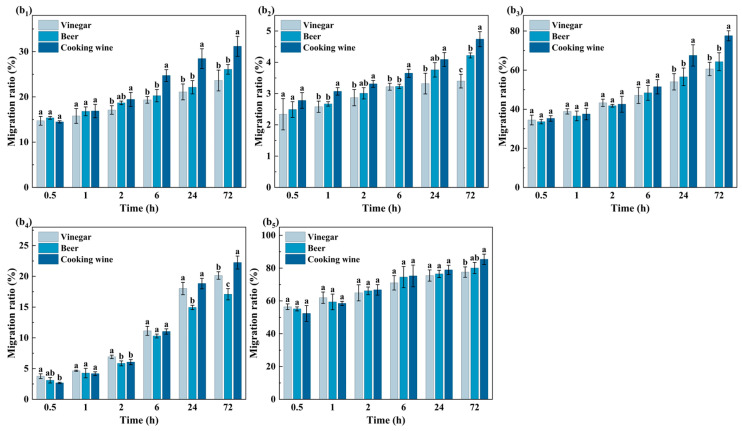
Migration ratios of five OPFRs from PP films into three acidic food systems (vinegar, beer, and cooking wine) at different contact times: (**b_1_**) TPhP, (**b_2_**) EHDPP, (**b_3_**) TBOEP, (**b_4_**) TnBP, and (**b_5_**) TPPO. Data is presented as mean ± standard deviation (n = 3). Different lowercase letters above the bars indicate statistically significant differences among different foods at the same contact time for the same compound (*p* < 0.05, Waller–Duncan test).

To explore the influence of acidic environment and ethanol on the migration behavior of acidic foods, the migration of five OPFRs in three typical acidic foods (vinegar, beer and cooking wine) was studied. As shown in Figure 3, in the early migration process (0.5–6 h), no apparent differences in five OPFRs’ migration ratios in three acidic foods were found, which meant the influence of different food matrices on the migration behavior could be neglected in this short period. However, in the late migration process (24–72 h), with the long contact time, the migration ratio of all four OPFRs except TPPO in cooking wine exceeded that in vinegar or beer. It was explained that the high ethanol content greatly enhanced the migration behavior by enhancing the interfacial mass transfer and permeation of OPFRs at the PP–food interface. In addition, the enhanced solubility also boosted the distribution opportunity of the compound in the system.

Interestingly, TPPO exhibited consistent migration behavior across all three acidic food matrices, unlike other OPFRs that showed matrix-dependent variations. This indicated that TPPO migration was fundamentally less influenced by the food matrix. In contrast, the matrix dependence of the migration behavior of TnBP and EHDPP was stronger. The slower migration ratio of TnBP in beer might be due to the higher soluble carbohydrate content, which might lead to hydrogen bonding or hydrophobic interactions with the alkyl side chains of TnBP and thus hinder its migration. EHDPP also consistently showed low migration behavior in vinegar, possibly due to the insufficient number of nonpolar components. In this case, the solubility of low-polarity and bulky molecules was limited, and therefore, migration into food from packaging materials was unlikely.

Generally, the migration process of OPFRs in acidic foods was affected by the combined effects of ethanol content and acidity. Higher ethanol content enhanced the diffusion and distribution of migrating substances between polymer and food matrix, while insufficient non-polar components had a certain inhibitory effect on the migration of low-polarity compounds. With the increase of organic content in the system, the effects of food matrix and OPFRs were more complicated, and it might have more influence on the migrating process.

#### 3.1.3. Migration Behavior Analysis of OPFRs in Dairy Products/Moderately Alcoholic Foods

**Figure 4:**
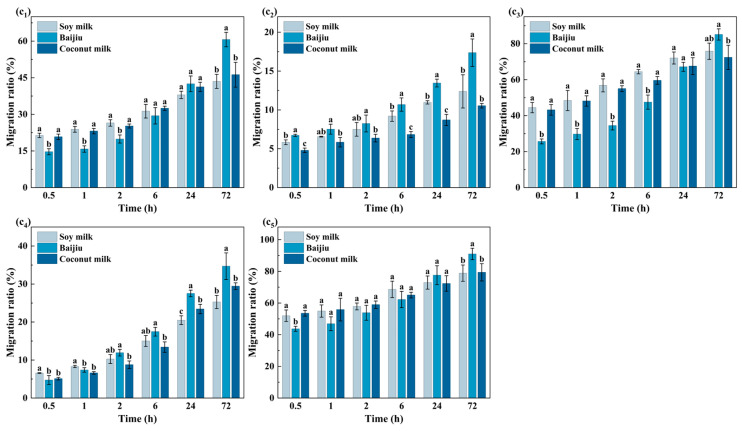
Migration ratios of five OPFRs from PP films into three dairy and medium-alcoholic food systems (soy milk, baijiu, and coconut milk) at different contact times: (**c_1_**) TPhP, (**c_2_**) EHDPP, (**c_3_**) TBOEP, (**c_4_**) TnBP, and (**c_5_**) TPPO. Data is presented as mean ± standard deviation (n = 3). Different lowercase letters above the bars indicate statistically significant differences among different foods at the same contact time for the same compound (*p* < 0.05, Waller–Duncan test).

To verify whether the increased organic content in the system further influences the migration behavior of OPFRs, this study analyzed the migration results of five OPFRs in soy milk, baijiu, and coconut milk. As shown in Figure 4, most OPFRs exhibited significant differences in migration ratios across various dairy products and moderately alcoholic beverages, indicating that food matrices exerted a significant impact on migration behavior. The migration ratio of TPPO remained stable across the three food matrices, and only at 72 h was it slightly higher in baijiu than in the other two foods, suggesting that the influence of the food matrix on its migration behavior was limited. The migration behavior of TBOEP exhibited distinct phased characteristics: during the initial migration phase, its migration ratio was elevated in soy milk and coconut milk due to enhanced solubilization by proteins and lipids. In the later migration phase, the interfacial mass transfer and permeation of OPFRs at the PP–liquor interface were gradually enhanced in the ethanol-rich system, leading to an increase in the migration ratio, which was eventually comparable to those observed in the other two food systems. The migration behavior of TPhP was like that of TBOEP. However, due to its lower polarity and lack of hydrophobic side chains, its interactions with proteins and lipids were limited. Consequently, in the later migration phase, its migration ratio in soy milk and coconut milk was significantly lower than that in baijiu.

Although TnBP possessed a long alkyl side chain, its limited interaction with proteins and lipids due to low molecular flexibility resulted in minor migration ratio differences among the three food samples during the initial migration phase. However, as ethanol-induced interfacial mass transfer and permeation at the PP–baijiu interface progressively intensified, the migration ratio in baijiu gradually increased, ultimately becoming significantly higher than in soy milk and coconut milk. EHDPP, characterized by the lowest polarity, larger volume, and poor flexibility, exhibited markedly restricted diffusion behavior in complex systems rich in lipids and proteins, such as soy milk and coconut milk, resulting in overall low migration levels. In contrast, the lower polarity of baijiu with its higher ethanol content allowed the hydrophobic side chains of EHDPP to form nonpolar interactions with ethanol molecules. This enhanced its solubility and partitioning capacity, significantly promoting migration behavior and resulting in the highest final migration level observed in baijiu.

In summary, the complex matrix components in dairy products and moderately alcoholic foods, including proteins, lipids and ethanol, promoted the overall migration of OPFRs through solubilization and enhanced interfacial mass transfer and permeation.

#### 3.1.4. Partition Coefficients and Diffusion Coefficients of OPFRs in Different Foods

To explore the equilibrium state and kinetic characteristics of OPFRs at the parameter level, this study quantitatively analyzed the migration behavior of OPFRs based on *K*_P/F_ and diffusion coefficient (*D*). The *K*_P/F_ primarily described the concentration and distribution of compounds when equilibrium is reached between the polymer and food, reflecting the migration amount under equilibrium conditions [41]. The *K*_P/F_ values were calculated according to the equilibrium relationship using Equation (4). As shown in Table 2, the calculated *K*_P/F_ exhibited a good correlation with relative migration capacity. This result indicated that variations in the *K*_P/F_ were closely related to the structural characteristics of the compounds. Compounds with higher molecular polarity and smaller volumes tended to migrate into more polar foods, resulting in relatively lower *K*_P/F_ values. Conversely, compounds with lower polarity and larger molecular volumes were more likely to remain in the hydrophobic polymer matrix, leading to relatively higher *K*_P/F_ values.

The diffusion coefficient (*D*) represented the diffusion flux under a unit concentration gradient, reflecting the rate at which the system reaches equilibrium during migration [42].

The value of *D* was obtained by fitting migration data using Equation (3). The diffusion coefficients obtained in this study were on the order of 10^−11^ cm^2^ s^−1^ and fell within the range reported by Li et al. for BHT, DBP and DEHP in PET at 40 °C [43]. The detailed results were presented in Table 2. The D values of compounds in different food types did not exhibit consistent patterns. Although several compound–food pairs showed relatively lower R^2^ values (0.827–0.845), the fitted curves still adequately described the overall migration trends and were therefore considered acceptable for the comparative analysis of *D*. Among them, EHDPP showed the highest *D* value in most foods, followed by TPPO, with TBOEP and TPhP at intermediate levels, while TnBP consistently had the lowest value. It was crucial to emphasize that a higher *D* value merely indicated a faster approach to equilibrium rather than a greater final migration. Therefore, a comprehensive analysis incorporating *K*_P/F_ and the initial loading in the polymer was essential for interpreting the final migration level. Taking EHDPP in aqueous foods as an example, its initial loading was identical to that of the other OPFRs, but both its *D* value and *K*_P/F_ value were markedly higher than those of the other OPFRs. Consequently, its migration behavior was characterized by a shorter time to reach equilibrium and a lower final migration level.

On the whole, *K*_P/F_ and *D* respectively reflected the equilibrium state and equilibrium attainment rate of OPFRs, jointly characterizing their migration behavior in different foods. However, migration behavior only reflects the extent to which compounds enter the food system and does not indicate their availability during digestion and absorption. Therefore, this study also analyzed the BA of OPFRs in different foods to quantify their proportion available during digestion.

### 3.2. Bioaccessibility of OPFRs in Different Foods

**Figure 5:**
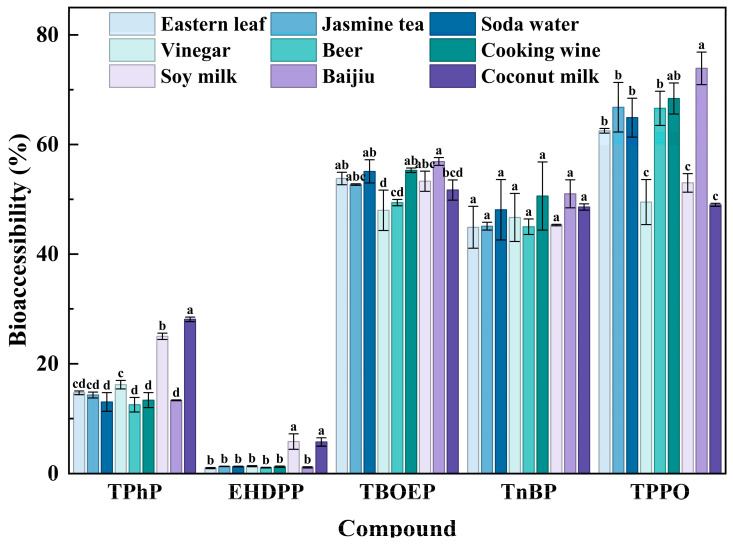
Bioaccessibility of five OPFRs in nine different food systems after in vitro digestion experiments. Data are presented as mean ± standard deviation (n = 3). Different lowercase letters above the bars indicate statistically significant differences among different foods for the same compound (*p* < 0.05, Waller–Duncan test).

To explore the impact of structural features and food matrices on the BA of OPFRs, the BA of five OPFRs in various foods was comprehensively studied based on the in vitro simulated digestion process. As shown in Figure 5, the BA of five OPFRs in different foods was well correlated with their polarity order: TPPO > TBOEP > TnBP > TPhP > EHDPP, which suggested that the molecular polarity was the main structural factor affecting the BA of OPFRs. Among them, the more hydrophobic TPhP and EHDPP exhibited significant BA variations across different food matrices, with notably higher BA in soy milk and coconut milk compared to other foods. This was attributed to the higher protein and lipid content in soy milk and coconut milk, which promoted the formation of mixed micelles, thereby enhancing their solubility and transport capacity in digestive fluids.

Contrarily, the BA of TPPO presented a different trend. TPPO exhibited higher BA in baijiu and cooking wine, with relatively medium levels in water-based food and beer, and the lowest levels in soy milk, coconut milk and vinegar. This could be mainly attributed to its high polarity: TPPO could not take advantage of the solubilization effects of lipids and proteins as much as the more hydrophobic TPhP and EHDPP. On the contrary, a higher ethanol content could greatly improve the solubility and dispersion characteristics of TnBP, leading to higher BA values in baijiu and cooking wine. The BA of TBOEP presented a similar trend to that of TPPO. However, the longer hydrophobic side chains of TBOEP resulted in higher BA values in soy milk and coconut milk due to its better interaction with proteins and lipids. Unlike the above compounds, the BA of TnBP varied little in different food items, and stayed at a moderately high level, which suggested low food matrix sensitivity.

In general, molecular polarity was the primary structural factor determining the BA ranking among compounds, while food matrix differences primarily exerted a regulatory effect, influencing the BA levels of the same compound across different foods. When combined with the migration results, compounds with higher migration levels did not necessarily exhibit higher BA.

### 3.3. Risk Assessment of OPFRs

Based on migration and in vitro simulated digestion experiments, this study calculated the EDI and HQ of five OPFRs in different foods using Equations (8) and (9), respectively, to assess their health risks. As shown in Table 3, although the absolute EDI values varied among different foods, the relative ranking of the five OPFRs within each food was generally consistent, exhibiting an overall ranking pattern of TPPO > TBOEP > TnBP > TPhP > EHDPP. Within the same food, the compound-specific EDI ranking was generally comparable to the corresponding migration ranking; however, noticeable differences were still observed for some compounds. The migration results showed that the migration ratio of TPhP was slightly higher than that of TnBP. According to a conventional exposure assessment based solely on migration data, the daily intake of TPhP would be expected to be comparable to that of TnBP. However, after incorporating BA, the EDI values of TnBP in all foods, except soy milk and coconut milk, were significantly higher than those of TPhP and were approximately twice those of TPhP. This indicated that, after accounting for BA, compounds with similar migration levels could exhibit clearly different EDI rankings, and that exposure assessment based solely on migration data could lead to biased exposure ranking.

Overall, EDI was jointly determined by migration, bioaccessibility and food consumption, and when comparing the same compound across different foods, food consumption often played a dominant role. Specifically, spirits, vinegar and cooking wine exhibited significantly lower overall EDI levels due to their lower daily consumption. In contrast, although water-based foods and beer showed relatively low migration and bioaccessibility levels, their overall EDI values were higher because of higher consumption. Soy milk and coconut milk exhibited the highest EDI values, which was attributed to their higher daily consumption as well as their higher migration and bioaccessibility levels.

When the addition level was 0.1 g/kg, the HQ values for all five OPFRs were below 1, indicating they fell within an acceptable risk range. Under high exposure scenarios, TBOEP and TPPO exhibited elevated HQ values of 1.51 × 10^−1^ and 1.78 × 10^−1^, respectively, due to their higher migration and BA levels coupled with lower RfD values. These compounds warranted particular attention. In contrast, although TnBP and TPhP also exhibited certain migration and BA levels, their relatively high RfD values resulted in lower HQ values and thus lower health risks. EHDPP exhibited both low migration and BA levels, resulting in HQ values consistently below 1 × 10^−4^—the lowest among the five OPFRs—indicating negligible health risks. Since RfD is a constant, HQ and EDI exhibit a linear relationship, meaning their distribution trends are identical within the same food product. Incorporating bioaccessibility into exposure assessment helped to avoid potential bias associated with relying solely on migration data and provided a more realistic estimation of internal exposure to OPFRs. The above low HQ values were obtained under a defined and relatively conservative additive level (0.1 g/kg) applied in the PP films, and actual additive levels and use conditions in practical applications may vary.

## 4. Conclusions

This study systematically compared the migration and bioaccessibility of five OPFRs from polypropylene packaging across different real food systems. The results showed that the relative migration ranking remained stable across matrices and temperatures and was primarily governed by diffusion behavior in the PP matrix, whereas food composition mainly influenced absolute migration levels. Bioaccessibility analysis further revealed that molecular polarity dominated BA, while matrix components such as ethanol, proteins, and lipids regulated inter-food differences. Incorporating migration and BA into exposure assessment demonstrated that similar migration levels could correspond to markedly different EDI rankings, indicating that migration alone did not characterize dietary exposure. At an addition level of 0.1 g/kg, all HQ values were below 1, although TBOEP and TPPO showed relatively higher values. Overall, integrating migration and BA provided a more realistic framework for evaluating packaging-related chemical exposure and supported additive screening and assessment.

One limitation of this study was that the migration experiments and exposure estimation were conducted at 40 °C with a surface-to-volume ratio of 6 dm^2^/L according to national guidelines, which might not represent real packaging geometries or higher-temperature use conditions. In addition, migration and in vitro digestion experiments were performed separately because the migrated concentrations in real foods were insufficient for direct bioaccessibility determination, limiting the linkage between these steps. In the future, risk assessment of OPFRs should be further explored in complex food matrices and under multivariate conditions.

## Figures and Tables

**Table 1:** Reference doses (RfDs) used for HQ calculation.

Compound	TPhP	EDHPP	TBOEP	TnBP	TPPO
RfD (ng/kg bw/day)	70,000	6000	15,000	10,000	20,000

RfD values used for HQ calculation were obtained from Li et al. (2025) for TPhP, EHDPP, TBOEP and TnBP, and from the U.S. EPA Provisional Peer-Reviewed Toxicity Values (PPRTV, accessed via PubChem) for TPPO [39].

**Table 2:** Fitted migration parameters of five OPFRs in nine foods.

	Compound	a	*K*_P/F_	D/(cm^2^·s^−1^)	R^2^
Eastern leaf	TPhP	0.328	762.51	8.00 × 10^−11^	0.845
	EHDPP	0.033	7681.78	9.59 × 10^−11^	0.93
	TBOEP	1.713	145.98	8.84 × 10^−11^	0.896
	TnBP	0.194	1290.98	5.16 × 10^−11^	0.914
	TPPO	3.293	75.92	9.53 × 10^−11^	0.928
Jasmine tea	TPhP	0.288	868.58	8.65 × 10^−11^	0.928
	EHDPP	0.036	7044.28	9.70 × 10^−11^	0.933
	TBOEP	1.33	187.98	8.77 × 10^−11^	0.929
	TnBP	0.159	1568.98	2.56 × 10^−11^	0.897
	TPPO	2.584	96.74	9.30 × 10^−11^	0.973
Soda water	TPhP	0.273	915.29	8.52 × 10^−11^	0.96
	EHDPP	0.033	7574.96	9.25 × 10^−11^	0.975
	TBOEP	1.537	162.68	8.81 × 10^−11^	0.956
	TnBP	0.204	1222.76	5.66 × 10^−11^	0.949
	TPPO	3.142	79.56	9.54 × 10^−11^	0.951
Vinegar	TPhP	0.309	803.3	8.99 × 10^−11^	0.924
	EHDPP	0.034	7098.21	1.07 × 10^−10^	0.976
	TBOEP	1.538	162.53	8.92 × 10^−11^	0.908
	TnBP	0.252	991.45	5.46 × 10^−11^	0.931
	TPPO	3.462	72.21	9.48 × 10^−11^	0.976
Beer	TPhP	0.353	708.45	8.95 × 10^−11^	0.858
	EHDPP	0.044	5681.07	8.84 × 10^−11^	0.893
	TBOEP	1.803	138.69	8.07 × 10^−11^	0.931
	TnBP	0.206	1212.84	5.48 × 10^−11^	0.944
	TPPO	4.013	62.3	9.26 × 10^−11^	0.975
Cooking wine	TPhP	0.453	552.05	7.04 × 10^−11^	0.98
	EHDPP	0.05	5024.95	9.10 × 10^−11^	0.871
	TBOEP	3.46	72.25	6.86 × 10^−11^	0.919
	TnBP	0.286	874.1	4.86 × 10^−11^	0.919
	TPPO	5.831	42.88	9.14 × 10^−11^	0.96
Soy milk	TPhP	0.771	324.39	7.67 × 10^−11^	0.923
	EHDPP	0.141	1768.35	7.39 × 10^−11^	0.956
	TBOEP	3.135	79.76	8.41 × 10^−11^	0.979
	TnBP	0.338	739.06	3.98 × 10^−11^	0.94
	TPPO	3.732	66.98	8.84 × 10^−11^	0.961
Baijiu	TPhP	1.541	162.21	3.61 × 10^−11^	0.889
	EHDPP	0.21	1189.25	6.50 × 10^−11^	0.875
	TBOEP	5.724	43.68	4.71 × 10^−11^	0.92
	TnBP	0.531	470.65	5.35 × 10^−11^	0.917
	TPPO	10.088	24.78	7.57 × 10^−11^	0.899
Coconut milk	TPhP	0.86	290.6	6.76 × 10^−11^	0.949
	EHDPP	0.118	2122.44	8.35 × 10^−11^	0.827
	TBOEP	2.623	95.31	8.87 × 10^−11^	0.948
	TnBP	0.417	599.53	2.23 × 10^−11^	0.881
	TPPO	3.868	64.63	9.18 × 10^−11^	0.897

**Table 3:** Estimated daily intake and hazard quotient of five OPFRs in Chinese adults.

	Median									
	EDI(ng/kg bw/day)					HQ				
Foods	**TPhP**	**EHDPP**	**TBOEP**	**TnBP**	**TPPO**	**TPhP**	**EHDPP**	**TBOEP**	**TnBP**	**TPPO**
Eastern leaf	96.79	0.85	905.78	194.25	1278.44	1.38 × 10^−3^	1.41 × 10^−4^	6.04 × 10^−2^	1.94 × 10^−2^	6.39 × 10^−2^
Jasmine tea	85.23	1.20	802.16	165.29	1284.33	1.22 × 10^−3^	2.00 × 10^−4^	5.35 × 10^−2^	1.65 × 10^−2^	6.42 × 10^−2^
Soda water	74.49	1.10	890.12	217.73	1312.87	1.06 × 10^−4^	1.83 × 10^−4^	5.93 × 10^−2^	2.18 × 10^−2^	6.56 × 10^−2^
Vinegar	145.08	9.61	538.78	152.67	557.34	1.82 × 10^−5^	2.55 × 10^−6^	6.46 × 10^−4^	3.13 × 10^−4^	6.40 × 10^−4^
Beer	16.16	0.40	96.87	35.38	134.47	9.34 × 10^−4^	1.52 × 10^−4^	4.24 × 10^−2^	1.54 × 10^−2^	5.33 × 10^−2^
Cooking wine	173.26	8.05	499.07	190.69	518.62	3.97 × 10^−5^	6.58 × 10^−6^	1.91 × 10^−3^	7.50 × 10^−4^	1.95 × 10^−3^
Soy milk	1.27	0.02	9.70	3.13	12.80	2.07 × 10^−3^	1.60 × 10^−3^	3.59 × 10^−2^	1.53 × 10^−2^	2.79 × 10^−2^
Baijiu	65.37	0.91	635.47	153.81	1066.29	2.31 × 10^−4^	6.60 × 10^−5^	6.46 × 10^−3^	3.54 × 10^−3^	6.72 × 10^−3^
Coconut milk	2.78	0.04	28.60	7.50	38.92	2.48 × 10^−3^	1.34 × 10^−3^	3.33 × 10^−2^	1.91 × 10^−2^	2.59 × 10^−2^
	95th									
	EDI(ng/kg bw/day)					HQ				
Foods	**TPhP**	**EHDPP**	**TBOEP**	**TnBP**	**TPPO**	**TPhP**	**EHDPP**	**TBOEP**	**TnBP**	**TPPO**
Eastern leaf	241.97	2.12	2264.44	485.62	3196.10	3.46 × 10^−3^	3.54 × 10^−4^	1.51 × 10^−1^	4.86 × 10^−2^	1.60 × 10^−1^
Jasmine tea	213.07	2.99	2005.41	413.24	3210.82	3.04 × 10^−3^	4.99 × 10^−4^	1.34 × 10^−1^	4.13 × 10^−2^	1.61 × 10^−1^
Soda water	186.22	2.75	2225.31	544.33	3282.18	2.66 × 10^−3^	4.58 × 10^−4^	1.48 × 10^−1^	5.44 × 10^−2^	1.64 × 10^−1^
Vinegar	435.24	28.84	1616.35	458.01	1672.01	7.28 × 10^−5^	1.02 × 10^−5^	2.59 × 10^−3^	1.25 × 10^−3^	2.56 × 10^−3^
Beer	48.47	1.19	290.62	106.15	403.41	3.11 × 10^−3^	5.06 × 10^−4^	1.41 × 10^−1^	5.13 × 10^−2^	1.78 × 10^−1^
Cooking wine	519.79	24.15	1497.20	572.08	1555.85	1.19 × 10^−4^	1.97 × 10^−5^	5.72 × 10^−3^	2.25 × 10^−3^	5.84 × 10^−3^
Soy milk	5.10	0.06	38.78	12.54	51.21	6.22 × 10^−3^	4.81 × 10^−3^	1.08 × 10^−1^	4.58 × 10^−2^	8.36 × 10^−2^
Baijiu	217.89	3.03	2118.22	512.70	3554.31	6.92 × 10^−4^	1.98 × 10^−4^	1.94 × 10^−2^	1.06 × 10^−2^	2.02 × 10^−2^
Coconut milk	8.33	0.12	85.80	22.51	116.77	7.43 × 10^−3^	4.03 × 10^−3^	9.98 × 10^−2^	5.72 × 10^−2^	7.78 × 10^−2^

## Data Availability

The original contributions presented in this study are included in the article. Further inquiries can be directed to the corresponding authors.
